# The development of emotional overeating: a longitudinal twin study from toddlerhood to early adolescence

**DOI:** 10.1186/s12966-025-01714-x

**Published:** 2025-02-10

**Authors:** Vaishnavi K. Madhavan, Zeynep Nas, Jacqueline Blissett, Clare Llewellyn, Moritz Herle

**Affiliations:** 1 Social, Genetic & Developmental Psychiatry Centre, Institute of Psychiatry, Psychology, and Neuroscience, King’s College London, London, UK; 2 Department of Biological Psychology, Vrije Universiteit Amsterdam, Amsterdam, The Netherlands; 3 Department of Behavioural Science & Health, University College London, London, UK; 4 School of Psychology & Institute of Health and Neurodevelopment, Aston University, Birmingham, UK

**Keywords:** Developmental psychology, Gemini twin study, Eating behaviour, Behaviour genetics

## Abstract

**Background:**

Previous research has estimated the genetic and environmental contribution to individual differences in emotional overeating in toddlerhood and early childhood. However, little is known how this behaviour tracks into adolescence. Here, we aimed to replicated previous work and examine the aetiology of stability and change in emotional overeating across time.

**Methods:**

Data were from the UK Gemini Twin Study, which includes 2402 twin pairs born in 2007. Parents reported on children’s emotional overeating at 16 months (*n* = 3784), 5 years (*n* = 2064), and 12 years (*n* = 964), using the Emotional Overeating Scale of the Child Eating Behaviour Questionnaire (CEBQ) at 5 and 12 years, and the CEBQ-T (toddler version) at 16 months. A Cholesky Decomposition twin model was used to quantify the additive genetic, shared, and nonshared environmental influences on emotional overeating at each time point, partitioned into aetiological effects unique to each age and those carried across time.

**Results:**

Additive genetic effects were minimal at 16 months and 5 years (9% and 7% respectively) but increased to 34% by 12 years. Shared environmental effects explained the majority of variance in emotional overeating at all three time points, but significantly less at 12 years (41%) than earlier (> 81%). The longitudinal phenotypic associations (*r* = 0.23–0.43) were explained by the shared environment.

**Conclusion:**

The shared environment plays a major role in the development of emotional overeating in early life. Most aetiological influences on emotional overeating were unique to each age, indicating the need for family-based interventions targeted to each developmental stage.

**Supplementary Information:**

The online version contains supplementary material available at 10.1186/s12966-025-01714-x.

## Introduction

Eating behaviours can be affected by negative emotions such as anger, fear, or sadness [1]. Emotional overeating (EOE) is the term used to describe the tendency to overeat in response to specific positive and negative emotions, and this phenomenon is most commonly studied in the context of negative emotions, such as sadness and stress. A recent systematic review and meta-analysis on eating behaviours in childhood and their impact on later weight found consistent associations linking EOE to higher weight in cross-sectional and prospective studies [2]. However, the relationship between EOE and weight is complex; not only has EOE been associated with higher BMI, but BMI itself has also been found to be a moderator between negative emotions and food intake– i.e. one study found that people living with obesity were more likely to increase their food intake in response to stress than those who had a healthy weight [3]. This might point towards a vulnerability among people with obesity to regulate their negative emotions using food. Subsequently, overeating and concerns about weight cause higher levels of distress in individuals with obesity, resulting in a potentially self-perpetuating cycle [4].

In addition to weight-related outcomes, EOE might also be a key behaviour in depression and eating disorders. Depression and obesity commonly co-occur, and longitudinal population-based studies in adults have suggested that emotional eating is one of the behavioural mechanisms linking the two [5]. Similarly, a comparison of emotional eating in patients with different eating disorders indicated that patients with bulimia nervosa and binge eating disorder had greater EOE in comparison to healthy controls, whereas, patients with anorexia nervosa had the lowest level of EOE [6]. Taken together, EOE has been proposed as an important behavioural intervention target for both obesity [7] and mental health [8].

Given the potential role of EOE in health outcomes, understanding its development is essential. Different theories have been proposed to explain how EOE develops, and all hypotheses so far have focused strongly on the role of learning. The psychosomatic theory [9] hypothesises that EOE arises from difficulty in distinguishing between the arousal caused by hunger versus that caused by negative emotions, which is the result of classical conditioning. It is speculated that parents who repeatedly soothe a child’s distress by offering food (so-called ‘emotional feeding’) leads to classical conditioning whereby the child is eventually cued to eat by a physiological stress response [10].

Operant conditioning may also contribute to the development of EOE through reduction or avoidance of negative emotion by eating of palatable foods. This can strongly reinforce learning to use food to escape from aversive internal states [1]. Experimental studies have shown greater emotional eating in laboratory settings in young children when their parents reported regular use of food to regulate children’s emotion, or who use food as a reward [11, 12], suggesting that EOE can be a taught-and-learned behaviour.

More recently, Chawner & Fillippetti [13] have conceptualised a developmental model for EOE, encompassing all processes and mechanisms that contribute to the development of EOE throughout infancy and childhood. They distinguish between two main factors influencing children’s probability to develop EOE - child characteristics (for example, child temperament and food approach behaviour such as food responsiveness and enjoyment of food) and environmental factors (most commonly parenting behaviours, in the case of young children). Furthermore, there is also evidence for the presence of complex interactions and reciprocal relationships between child characteristics, parental factors, and EOE [11, 14–16]. To add to the complexity, these interrelations can also have differential influences at different developmental stages.

Together these studies suggest that the early feeding environment shapes the development of EOE, but the degree to which nature and nurture influence this developing phenotype over time is not yet understood.

The extent to which phenotypes are influenced by genetic and environmental influences can be elucidated by behavioural genetic designs. A powerful method are twin studies, which separate individual differences into additive genetic effects, also known as heritability (A), shared environmental influences (those shared entirely by co-twins, and contribute to twin similarity, such a parental emotional eating) (C), and the nonshared environmental influences (aspects of the environmental/unique experiences that are not shared by co-twins, making them different, such as one twin experiencing weight-related bullying) (E) [17].

So far, three twin studies on EOE using adult samples and two using children have been published. Using data from the Gemini Twin Study, Herle et al. [18] estimated the heritability of EOE at 10% at 16 months and 4% at 5 years of age. These findings were broadly replicated in smaller sample of 4-year-old British twins obtained from the Twins Early Development Study (TEDS) [19]. Contrastingly, moderate genetic effects were found for emotional eating in adult samples, with estimates ranging from 25 to 60% across samples [20, 21]. As expected, the shared environment of the twins had the biggest influence in toddlerhood (88%) and childhood (93%) with only small effects from the nonshared environmental factors at this very young age [18, 19]. whereas during adulthood, the nonshared environment contributed far more to variation in EOE than the shared environment.

In the current study, we attempt to build on the previous findings in this sample [18], by examining the longitudinal changes in EOE up to early adolescence at 12 years beyond toddlerhood at 16 months and early childhood at 5 years. This longitudinal study design offers a significant advantage as it contributes to the understanding of relative changes in genetic, shared, and nonshared environmental influences on EOE as children age. Previous studies using this design to investigate other behavioural phenotypes, commonly find that the genetic contribution increases with age [18]. This is often attributed to the fact that with age children’s autonomy increases, allowing them to make their own choices and spend more time with peers. Hence, in the context of EOE we hypothesise an increase of genetic contributions in early adolescence. This paper used data from a population-based UK twin birth cohort to address this outstanding gap in the literature by quantifying the impact of genetic and environmental influences on EOE across three key time periods– 16 months (toddlerhood), 5 years (early childhood), and 12 years (early adolescence). In addition, we also studied the stability and change in aetiological factors across these three developmental periods.

## Method

### Participants

The analysis sample was drawn from Gemini, a population-based UK twin birth cohort (http://www.geministudy.co.uk/). Gemini was established to examine the genetic and environmental influences on weight trajectories in early childhood, with detailed and repeated measures of children’s appetite, food preferences, activity, and parental feeding styles at important developmental timepoints in early life, and later childhood [22].

Families with twins born in England and Wales between March and December 2007 were contacted for participation by the Office for National Statistics. Of *N* = 6754 eligible families, *n* = 3435 families consented to be contacted by the research team for participation (51% of all eligible families). Of these, *n* = 2402 families consented to take part in the study and completed the baseline questionnaire (70% of those invited to take part; 36% of all eligible families). The baseline sample included *n* = 749 monozygotic pairs (MZs), *n* = 1616 dizygotic pairs (DZs), and *n* = 37 pairs of unknown zygosity. Compared with national twin statistics, the baseline sample was generally representative of twins in the UK in terms of zygosity, sex, gestational age at birth, and birth weight [22]. Follow-up data were collected at key points of development. Emotional overeating (EOE) was measured at 16 months (*n* = 3784), 5 years (*n* = 2064), and 12 years (*n* = 964). To be included in the current analysis, the sample of twins needed to have data on sex, zygosity, age and EOE score for at least one of the three time points (*n* = 3882 individual twin children). Ethical approval for the Gemini study was granted by the University College London Committee for the Ethics of non–National Health Service Human Research.

### Materials

#### Child eating behaviour questionnaire (CEBQ)

Emotional Overeating was measured using the Child Eating Behaviour Questionnaire (CEBQ) [23], a parent-reported tool developed to quantify eight different eating behaviours with a total of 35 items. In the current study, only scores from the Emotional Overeating (EOE) subscale were used, which consists of 4 items, each scored on a 5-point Likert scale ranging from ‘Never’ to ‘Always’. The EOE subscale shows high internal reliability (α = 0.72–0.79) and moderate correlation between scores over a two-week period (*r* = 0.52) [20].

EOE was measured at three time points– 16 months, 5 years, and 12 years. At 5 years and 12 years, the standard CEBQ scale was used consisting of the following four items: “My child eats more when worried”, “My child eats more when annoyed”, “My child eats more when anxious”, and “My child eats more when s/he has nothing else to do.” At 16 months, the Child Eating Behaviour Questionnaire– Toddler version [18] was used, which was modified to ensure that the emotion adjectives used in the scale are age-appropriate. For example, ‘worried’ was changed into ‘irritable’, ‘annoyed’ into ‘grumpy’ and ‘anxious’ into ‘upset’. The fourth item “my child eats more when s/he has nothing else to do” was omitted from the toddler version of the scale since pilot qualitative work with a sample of mothers of 18-month-old toddlers revealed that this behaviour cannot be observed at such a young age. The toddler version, therefore, contains only 3 items. The mean of the item scores were calculated; at each age, scores were included in the analysis if a minimum of 2/3 (16 months) or 2/4 items (5 and 12 years) were completed.

#### Demographic information

The sex of each twin was parent-reported at baseline. The age of the twin at time of measurement of EOE was calculated from parent-reported date of birth provided at baseline, and the date of completion of questionnaire. Zygosity was established by using the following method: (i) opposite-sex twins were automatically categorised as ‘dizygotic’; the zygosity of same-sex twins was established using a validated 20-item questionnaire, reported by parents at baseline and 29 months; (iii) DNA testing of pairs who could not be assigned using the zygosity questionnaire; and (iv) DNA validation of questionnaire assigned pairs using random sampling, with 100% agreement in a random sample of *n* = 81 pairs (43 MZ twins and 38 DZ twins) at 29 months of age [24].

### Statistical analysis

#### Data preparation

Twins with unknown zygosity were excluded from analyses (*n* = 37 pairs). EOE scores were regressed on sex and age of the twins at the time of measurement to reduce inflation of shared environmental estimates (as age is correlated completely for all pairs, and sex is correlated for all same-sex pairs).

##### Phenotypic correlations

Longitudinal EOE correlations across toddlerhood (16 months), early childhood (5 years) and early adolescence (12 years) were estimated to establish continuity in EOE over this developmental period.

##### Twin model fitting

The principle underlying twin analyses is that MZ twins share 100% of their segregating genes, whereas DZ twins share, on average, 50% of their segregating genes. Importantly, it is assumed that both members of a twin pair share their environment to the same extent (e.g., raised in the same household, same parents), and so any differences in the magnitude of correlations between MZ and DZ pairs are attributable to the influence of genetics. If the difference in resemblance between MZ and DZ twins is high, then individual differences in an observed behaviour are more likely to be shaped by genetic differences. In contrast, if the MZ and DZ twin pairs resemble each other to a similar degree, this is an indication of the influence of shared environmental factors. Any variance that cannot be attributed to shared environmental and genetic components, is explained non-shared environmental effects (any environmental factor that results in twin pair difference) and measurement error [17]. This concept can be also applied to longitudinal data estimating the genetic and environmental influences on the longitudinal association between the twins. For example, the correlation between the EOE scores of twin 1 at 16 months and the scores of twin 2 at 5 years is calculated, for MZ and DZ pairs separately. MZ and DZ correlations are compared, which provides an estimate of the continuing contribution of genetic, shared, and unique environmental influences from one time period to the next. If the MZ-DZ correlations resemble a 2:1 ratio, this would indicate that genetic factors are more likely to explain the variance at that time point, whereas a 1:1 ratio would indicate that shared environmental effects are more influential.

##### Multivariate ACE model– cholesky decomposition

**Fig. 1:**
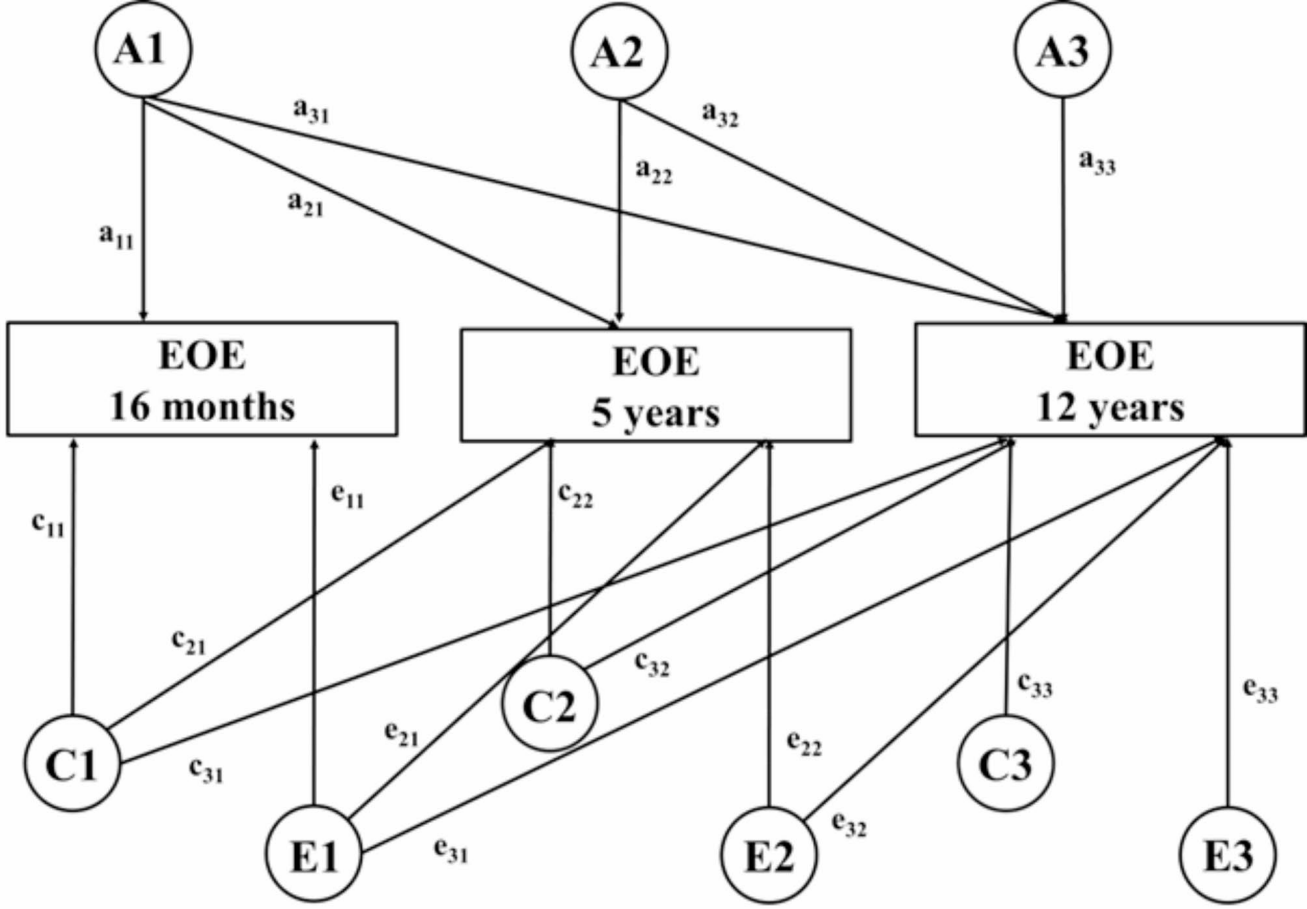
Multivariate cholesky decomposition model Figure 1 represents the multivariate Cholesky decomposition model. The rectangular boxes represent the phenotype EOE (Emotional Over-Eating), measured at three time points– 16 months, 5 years, and 12 years. The latent factors of additive genetic effects (A), shared environmental effects (C), and non-shared environmental effects (E) are represented within circles. The unique contribution of A, C, and E at each time point is represented by A1, C1, E1, A2, C2, and so on. The effects carried over from one time point to another is denoted by a11, a21, a31, c11, and so on

A longitudinal ACE Cholesky Decomposition model (see Fig. 1) was used to derive estimates of additive genetic effects (A), shared environmental effects (C) and nonshared environmental effects (E) at each time point– 16 months, 5 years, and 12 years (denoted by A1, C1, E1, for timepoint 1; A2, C2, E2 for timepoint 2; and A3, C3 and E3 for timepoint 3). Parameter estimates of A, C and E at each time point were obtained with 95% confidence intervals. In addition, the overlapping effects of A, C, and E carried over from one time point to another (denoted by a11, a21, a31, c11, and so on) were also estimated. This measure indicates the extent to which genetic, shared, and nonshared environmental effects on EOE at 16 months is the same as those at 5 years and at 12 years. A high value of a21, for example, would indicate that the majority of additive genetic effects on EOE at 16 months also influences EOE at 5 years.

Prior to fitting the longitudinal ACE Cholesky Decomposition model, we fitted an unconstrained fully saturated model to create a baseline, and two sub models that test some of the assumptions of twin analyses. Model fit is assessed with minus twice the log likelihood (− 2LL). Difference in − 2LL between a fully saturated model and a nested sub model (simpler model with fewer parameters) was assessed by χ2 tests and p-values. The more parsimonious nested model is preferred if this does not result in a significant reduction in fit. In addition, we used the Akaike Information Criterion (AIC) with lower values indicating the better fitting model [25]. Sub model 1a is a constrained model which assumes equal means and variances across twin order, whereas sub model 2a assumes equal mean and variances across twin order as well as zygosity groups. Sub model 1a was compared to the fully saturated model and sub model 1b was compared to sub model 1a. Both tests were found to have non-significant p-values (sub model 1a, AIC = -9648.525, *p* = 0.19; sub model 1b, AIC = -9586.844, *p* = 0.54), indicating that the constrained models with fewer parameters fitted the data well. A full list of fit statistics for all models can be found in Table 1.

**Table 1:** Fit statistics for the different models

Model No.	Model Name	Compared to Model	ep	df	-2LL	AIC	Δχ²	*p*-value
1	Fully saturated model	-	54	6744	-5435.711	-5327.711	-	-
2	Sub model 1a	1	42	6756	-5411.441	-5327.441	12	0.19
3	Sub model 1b	2	21	6777	-5389.997	-5347.997	21	0.43
4	Full ACE Cholesky Decomposition	1	21	6777	-5389.997	-5347.997	33	0.07

**Abbreviations**: ep: estimated parameters, df: degrees of freedom, -2LL: -2 Log Likelihood, AIC: Akaike’s Information Criterion, Δχ²: difference in chi-squared. Sub model 1a: Constrained model which assumes equal means and variances across twin order; Sub model 1b: Constrained Model assumes equal mean and variances across twin order as well as zygosity groups

All statistical analysis was carried out in RStudio, using the OpenMx package [26]. Analyses code is available at https://github.com/MoritzHerle/Gemini_EOE.

## Results

### Descriptive statistics

Sample characteristics are shown in Table 2. EOE scores were available for *n* = 3784 at 16 months, *n* = 2064 at 5 years, and *n* = 964 at 12 years. The total sample included 3882 twins, who had baseline measurements all of age, sex, and zygosity, as well as EOE data on at least one of the three time points. The sample used in this analysis did not differ from the full baseline sample in terms of zygosity, sex, gestational age, or birth weight. Mothers who provided at least one measure of emotional eating were older at birth in comparison to mothers at baseline. A full list of comparisons between the sample at baseline and the analyses sample can be found in Supplement Table 2.

**Table 2:** Sample characteristics at baseline, 16 months, 5 years, and 12 years

	Baseline *N*(%) Mean (SD)	16 months *N*(%) Mean (SD)	5 years *N*(%) Mean (SD)	12 years *N*(%) Mean (SD)
**Sample size with EOE scores (n)**	4804	3784	2064	964
**Age (months or years)**	-	15.8 (0.1)	5.15 (0.13)	12.69 (0.43)
**Sex assigned at birth (males)**	2386 (49.67%)	1862 (49.21%)	1009 (48.89%)	472 (48.96%)
**Zygosity (monozygotic twins)**	1498 (31.18%)	1228 (32.45%)	696 (33.72%)	334 (34.65%)
**Gestational Age (in weeks)**	36.2 (2.48)	36.21 **(**2.46)	36.26 (2.43)	36.32 **(**2.53)
**Weight at Birth (in kg)**	2.46 (0.54)	2.47 (0.54)	2.46 (0.54)	2.47 **(**0.53)
**Maternal Age at Birth**	32.95 (5.19)	33.37 (5.04)	33.84 (4.74)	34.23 (4.37)
**Ethnicity (White)**	4178 (86.99%)	4114 (88.42%)	1850 (89.63%)	872 (90.46%)
**EOE Score (SD)**	-	1.64 (0.59)	1.57 (0.51)	1.75 (0.61)

Abbreviations: EOE: Emotional overeating, SD: Standard deviation

### Phenotypic correlations (within twin cross time)

Table 3 lists the phenotypic (longitudinal) correlations between 16 months, 5 years, and 12 years for EOE, separated by MZ and DZ twins as well as within and across twin pairs. Within twin across time correlations were small to moderate in size, suggesting that twins who engaged in emotional overeating in toddlerhood tended also to overeat in response to negative emotions in childhood and in adolescence, to some extent. The association between 5 and 12 years was larger, indicating that stability in EOE may be stronger from early childhood to adolescence.

**Table 3:** Intraclass correlations and the cross-twin-cross-time correlations for monozygotic and dizygotic twins with 95% CIs

Monozygotic twins (MZ)							
		Twin 1			Twin 2		
		16 m	5y	12y	16 m	5y	12y
**Twin 1**	**16 m**	1					
	**5y**	0.28 (0.22, 0.33)	1				
	**12 y**	0.22 (0.14, 0.29)	0.43 (0.36, 0.50)	1			
**Twin 2**	**16 m**	0.97 (0.97, 0.97)	0.28 (0.22, 0.33)	0.22 (0.14, 0.29)	1		
	**5y**	0.28 (0.22, 0.33)	0.98 (0.97, 0.98)	0.43 (0.36, 0.49)	0.28 (0.22, 0.33)	1	
	**12 y**	0.22 (0.14, 0.29)	0.43 (0.36, 0.49)	0.95 (0.93, 0.96)	0.22 (0.14, 0.29)	0.43 (0.36, 0.50)	1
**Dizygotic Twins (DZ)**							
		**Twin 1**			**Twin 2**		
		**16 m**	**5y**	**12 y**	**16 m**	**5y**	**12 y**
**Twin 1**	**16 m**	1					
	**5y**	0.28 (0.22, 0.33)	1				
	**12 y**	0.22 (0.14, 0.29)	0.43 (0.36, 0.50)	1			
**Twin 2**	**16 m**	0.92 (0.92, 0.93)	0.28 (0.23, 0.34)	0.23 (0.15, 0.30)	1		
	**5y**	0.28 (0.23, 0.34)	0.93 (0.92, 0.94)	0.40 (0.32, 0.47)	0.28 (0.22, 0.33)	1	
	**12 y**	0.23 (0.15, 0.30)	0.40 (0.32, 0.47)	0.73 (0.67, 0.77)	0.22 (0.14, 0.29)	0.43 (0.36, 0.50)	1

**Abbreviations**: MZ: Monozygotic, DZ: Dizygotic, 16 m: 16 months, 5y: 5 years, 12y: 12 years

### Twin correlations

#### Univariate twin (cross-twin, within-time) & longitudinal twin correlations (cross-twin, cross-time)

Estimates for all twin correlations for MZ and DZ twins are presented in Table 3. MZ and DZ correlations between the twins are relatively high and similar at 16 months (MZ = 0.97, DZ = 0.92) and 5 years (MZ = 0.98, DZ = 0.93), but twin pair differences had increased by age 12 years (MZ = 0.95, DZ = 0.73). The longitudinal cross-twin-cross-time (CT-CT) correlations between 16 months and 5 years were relatively low and similar for MZ and DZ twins; the correlation between Twin 1 at 16 months and Twin 2 at 5 years (and vice versa) for MZ and DZ twins was found to be 0.28. This indicates that the longitudinal association between EOE at 16 months and 5 years is mostly driven by continuing shared environmental influences, although only to a small extent. A similar trend was observed for the CT-CT correlations between 16 months and 12 years (MZ Twin 1 at 16 months and MZ Twin 2 at 12 years = 0.22, DZ Twin 1 at 16 months and DZ Twin 2 at 12 years = 0.23), which indicated by shared environmental influences, but to a lesser extent.

### Cholesky decomposition

**Fig. 2:**
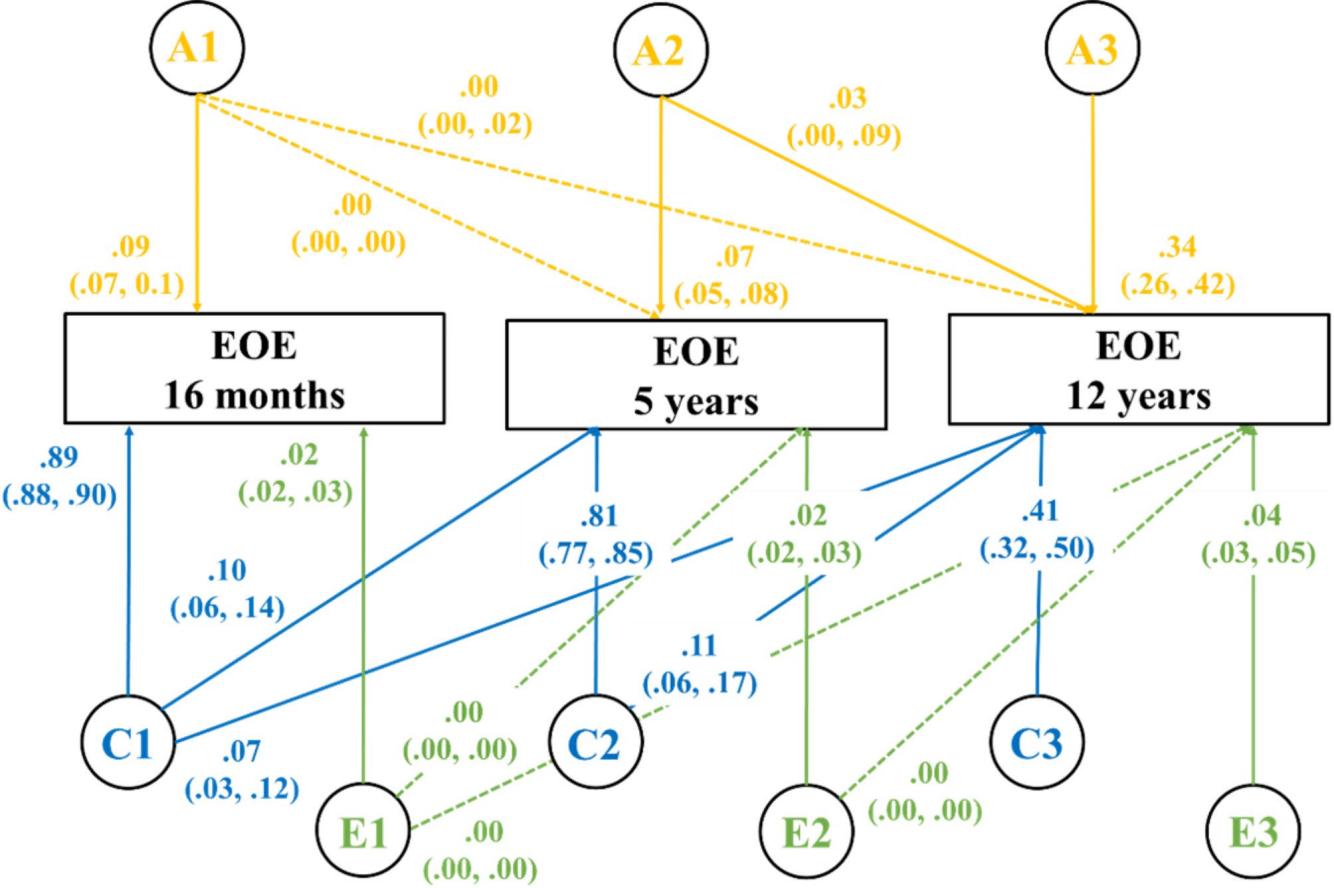
Cholesky decomposition with the estimates of A, C, and E on EOE across childhood The rectangular boxes represent the phenotype Emotional Overeating (EOE) at three time points– 16 months, 5 years, and 12 years. The latent factors are represented with circles and provide the estimates of additive genetic effects (A), shared environmental effects (C), and non-shared environmental effects on EOE. The lines indicate the contribution of A, C, and E at each time point as well as the carry-over effects to the successive time points. Estimates presented are standardised. The solid and dotted lines represent significant and non-significant influence of the latent factors to EOE respectively

The longitudinal Cholesky decomposition ACE model fitted the data well (Table 1). The results are shown in Fig. 2. Additive genetic effects (A) on EOE were found to be minimal at 16 months and 5 years (9% and 7% respectively) but increased to explain 34% of the variance at 12 years indicating a moderate genetic influence. The shared environment had the greatest influence on EOE across the three time points, contributing 89% and 81% to the variance in EOE at 16 months and 5 years respectively and although reduced, continued to explain 41% of the variance at 12 years. The shared environmental influence decreased significantly from 16 months to 5 years and again from 5 years to 12 years of age. The unique environmental effect estimates ranged 2–4%, indicating minimal and negligible influence of nonshared environmental factors on EOE through toddlerhood and childhood.

Additive genetic effects on EOE at 16 months did not carry over to 5 or 12 years, as indicated by the non-significant paths from A1 to EOE at 5 years (*r* = 0.00, 95% CI: 0.00, 0.00) and 12 years (*r* = 0.00, 95% CI: 0.00, 0.02). EOE at 5 years and 12 years the share of additive genetic effects was close to zero (0.03, 95% CI: 0.00, 0.09). Of the shared environmental influences at 16 months, 7% (95% CI: 0.03, 0.12) and 10% (95% CI: 0.06, 0.14) continued to influence EOE at 5 years and 12 years respectively. Moreover, 11% (95% CI: 0.06, 0.17) of the shared environmental factors at 5 years are carried over to 12 years. Overall, the shared environment of the twins seems to differ significantly at each time point, with only very minimal factors carried over from the previous time points. In terms of the nonshared environment, all three paths from 16 months to 5 years and 12 years, and from 5 years to 12 years were found to be non-significant. This shows that none of the non-shared environmental factors influencing EOE at one time point impact EOE at the successive time points.

## Discussion

The current study examines the relative contribution of genetic and environmental influences on EOE from toddlerhood to adolescence, and stability and change in influences over time. Aspects of the environment shared entirely by co-twins exerted the greatest influence on individual differences in EOE at all three time points, although it decreased significantly over time, explaining 89% of the variance at 16 months, 81% at 5 years, and 41% at 12 years. Genetic influences were significant although relatively low during toddlerhood and early childhood (contributing 9% and 7% respectively), but by 12 years, the genetic contribution to EOE had risen to 34%, indicating a sizeable influence in early adolescence.

Our findings build on a previous study in this sample [18], which estimated minimal additive genetic effects on EOE as 10% at 16 months and 5% at 5 years. Extending these results to early adolescence, we find that genes explained 34% of the variance in EOE at 12 years, an increase from estimates observed during toddlerhood and early childhood. The heritability estimates seem to be comparable to the genetic effects found in adult samples in Korea [27] and Finland [21], and higher than estimates found in other adult samples in Sweden and the UK [20, 21]. Overall, these results support environmental developmental theories of EOE, which propose that this a largely learned behaviour. Specially, the psychosomatic theory suggests that external input, conditioned through parental feeding behaviour, leads to difficulties in distinguishing between the arousal caused by negative emotions versus arousal caused by hunger [9, 10].

However, it is difficult to compare the results of the current study with those from adult twin studies due to major methodological variation. For adult samples, tools like the Three Factor Eating Questionnaire (TFEQ-18) [28] and Dutch Eating Behaviour Questionnaire (DEBQ) [29] are often used, which are self-report questionnaires measuring emotional eating, as opposed to the CEBQ used in the current study, which is a parent-reported tool assessing EOE.

In our study, EOE was found to track moderately from toddlerhood through early childhood and early adolescence. This longitudinal association through all three time points was entirely explained by the continuing shared environmental influences, as there were no continuing genetic or non-shared environmental influences. However, the proportion of the total shared environmental influences at each that were common across other ages was small: 10% (95% CI: 0.06, 0.14) and 7% (95% CI: 0.03, 0.12) of C at 16 months influenced EOE at 5 years and 12 years respectively; similarly, 11% (95% CI: 0.06, 0.17) of the variance in EOE at 12 years was explained by the shared environmental influences carried over from 5 years. The above findings indicate that most shared environmental influences newly emerge and are therefore unique to each time point. It is reasonable to conclude that EOE during toddlerhood, early childhood, and adolescence is largely influenced by novel environmental (and genetic, at age 12) factors arising at each time point. This seems reasonable, if we consider parental feeding behaviours as one of the main drivers of the development of EOE in childhood. As children grow up and become more independent, the control that parents have over their child eating behaviour and food environment decreases substantially. This gained independence can lead to children being free to act on their genetic propensities, resulting in increases in estimated heritability. Similarly, as the impact of the shared environment decreases, influences from the non-shared environment (such as twin specific peer groups etc.) tend to increase as well. These particular insights can only have been gained from using a longitudinal twin design, which tracks the genetic and environmental contributions to individual differences in EOE across time. When considering preventative efforts, these results suggest, as overall shared environmental influence decreases with age, that potential interventions might be better placed to focus on toddlerhood rather than later in development.

The results of the study underscore the substantial influence of the early environment on the development of EOE. By targeting and addressing modifiable external factors that contribute to EOE, there is a potential for significant impact in the context of obesity prevention, given the link between EOE and childhood adiposity [2]. However, twin studies only offer an estimate of the relative contribution of the early environment on EOE, they do not specify the factors at play. Nonetheless, research into the specific environmental factors influencing EOE and their relative influence in the development of maladaptive eating behaviour can inform preventative public health interventions. In addition to environmental factors, results suggested that genetic influence explained 34% of the variance at 12 years, which is considerably higher than the estimates during toddlerhood and early childhood.

Parental feeding practices are an example of the shared environmental factors that influence EOE. In a Norwegian sample of 797 children, instrumental feeding practices, wherein the parent uses food as a reward at age 6 was associated with increases in EOE from age 6 to 8 [30]. Other studies have found significant associations between EOE and emotional feeding [31, 32]. Similarly, excessively controlling the child’s food intake [15, 33] and modelling emotional overeating behaviour are also parental feeding practices that have been linked to EOE in children [34]. More recently, we showed, using data from the Gemini Twin Study, that instrumental and emotional feeding in toddlerhood was associated with later EOE and vice versa, pointing towards a complex cascading feedback loop between child eating behaviour and parental feeding behaviours [35]. Interventions focused on facilitating responsive and developmentally appropriate feeding practices have been shown to have a positive influence on EOE observed in children, which provides evidence for parental feeding as a key factor in the development of EOE. One example is a randomised controlled trial (INSIGHT) examining the effect of a responsive parenting intervention on feeding practices and eating behaviour in infants. In comparison to the control group, the mothers reported lower use of emotional feeding and perceived the infant to exhibit lower levels of EOE [36]. Similar results were found in the NOURISH trial, which also found lower EOE following a parenting intervention in 2-year-olds, however this effect did not extent to later follow-up points [12, 37]. These studies provide initial insight into the role of interventions in modifying the early environment to change emotional overeating behaviour and potentially weight outcomes in children.

In addition to parental feeding practices, other household factors have also been implicated in the development of EOE. For instance, stressful living situations early in life has been linked to obesity, possibly because children learn to cope with stressors by eating [38]. Stress in the home may therefore (inadvertently) make the environment more conducive to the development of emotional eating behaviours, resulting in later higher weight. Indeed, household chaos has been associated with emotional eating in children as young as 24 months [39]. Understanding the role of such early environmental factors shaping EOE provides valuable insights for public health interventions and underscores the importance of creating supportive and nurturing environments for promoting the development of healthy eating habits early in life.

This study is the first to indicate that most shared environmental influences newly emerge and are therefore unique to each time point. Emerging adolescence is an important developmental period, bringing its own set of specific risk factors. For example negative body image [40], adverse life events [41], and smartphone usage [42] have been shown to influence EOE in adolescence, which are distinctly different from the ones influencing EOE in childhood. In addition, these are the first results suggesting that even though there is a dominant influence of the shared environment, heritability estimates of EOE substantially increase with age. This phenomenon might be explained by gene-environment correlation [43]. Genetic influences are expressed more when individuals have the agency to make decisions and act out their innate predispositions. Consequently, genetic influences may be minimal in early life because of young children’s lack of agency over their food choices, and their inability to consciously use food to regulate their emotions. As the child grows up and becomes increasingly autonomous, it is expected that heritability estimates increase and are high during adulthood, as evidenced by literature on EOE.

### Limitations

The study has several strengths including data from a large-scale population-based cohort, prospective design, and longitudinal examination of EOE from toddlerhood through early adolescence. Gemini twins are representative of the wider UK population in terms of characteristics such as zygosity, sex, gestational age at birth, and birth weight. However, the sample has a high maternal age and overrepresentation of White families. Even at baseline, the sample consisted of 87% White families, and this proportion increased slightly over time. This limits the generalisability of the results to the wider population.

This overrepresentation of White families in the sample may be particularly problematic in the case of investigating eating behaviours in young children. This is because different ethnic groups adopt varied feeding practices based on their cultural beliefs and values. In the UK, South Asian and Black Afro-Caribbean parents have been shown to exhibit significantly different feeding practices in comparison with White families, using higher authoritarian, non-nutritive, and emotional feeding practices [44]. Therefore, the overrepresentation of White families in the current sample would not fully capture the eating behaviours from diverse ethnic backgrounds.

Moreover, one key concern in twin studies is the generalizability of the findings to the general population, particularly to singletons, who may have significantly different childhood experiences in comparison to twins. For example, families with twins and singletons may differ significantly in terms of socioeconomic strata, parenting styles, family structures and so on [45]. There are also concerns regarding the violation of the ‘equal environments assumption’ in twin studies, which assumes that MZ and DZ twins share their environments to the same extent. However, both of these arguments have been refuted by several authors [46, 47], and a misclassified zygosity design validated the use of parent rated eating behaviour questionnaires in this twin sample [24].

Average scores of EOE were relatively low at all three time points. It is reasonable to conclude that emotional overeating may not be very common in early life since the children do not actively seek out food to regulate their negative emotions. Previous studies conducted on toddlers between the ages of 12–24 months seem to have reported similarly low scores of EOE using the CEBQ [48–50]. Alternatively, the relatively low scores of EOE may be reflective of the limitations associated with a parent-rated tool, in that parents might not be able to identify signs of EOE in their children. Emotional eating can be difficult to identify in another individual since an observer may not have reliable knowledge regarding another person’s internal emotional states and motivations at the time of eating. Further, the CEBQ assumes that all emotions included on the scale have an equal effect on the child’s appetite, whereas it is possible that some might have a bigger impact than others. Future research might consider studying how specific emotions (i.e. sadness versus distress) might have differential effects on desire to eat.

Nevertheless, psychometric tools such as the CEBQ remain the most feasible option to measure eating behaviour in large numbers of children. Observational measurements, often rated by an independent observer, offer many advantages, but are too resource- and time-intensive to use in population-based twin studies, which require a large sample size and, sometimes, long-term follow-up. Moreover, mothers are likely to be a good judge of their children’s behaviour, since they typically spend a significant amount of time in caretaking and may be more aware of their children’s emotional states and their typical responses.

## Conclusion

The present study found that the shared environment of twins plays a major role in the development of EOE during toddlerhood and early childhood. On the other hand, genetic factors only play a minimal role early in life but increase to contribute moderately to EOE in adolescence. These results support developmental theories of EOE, which propose that this behaviour is largely learned during childhood. Furthermore, we propose that interventions to support parents should target toddlerhood rather than mid childhood, as our findings suggest this as most promising time period.

## Electronic supplementary material

Below is the link to the electronic supplementary material.

Supplementary Material 1

Supplementary Material 2

## Data Availability

The datasets used and/or analysed during the current study are available from the corresponding author on reasonable request.

## Abbreviations

EOE: Emotional overeating
BMI: Body Mass Index
CEBQ: Child Eating Behaviour Questionnaire
MZ: Monozygotic
DZ: Dizygotic
AIC: Akaike Information Criterion
Df: Degrees of freedom
Ep: Estimated parameters
