# Supplementary material for: The development of emotional overeating: a longitudinal twin study from toddlerhood to early adolescence
Source: Int J Behav Nutr Phys Act. 2025 Feb 10;22:17. doi: 10.1186/s12966-025-01714-x (PMC11812261; doi:10.1186/s12966-025-01714-x)
Supplement: Supplementary file 2 — Supplementary Material 2 [file 12966_2025_1714_MOESM2_ESM.docx]

Supplementary material

**Supplement table 1.** Non-response analyses, comparisons between sample at baseline and analyses sample (with at least one measurement of emotional overeating)

|  | **Sample at baselines**  N(%) or means(SD) | **Analyses sample**  N(%) or means(SD) | **Ch-square value/**  **t-value** | **Degrees of freedom** | **P-value** |
| --- | --- | --- | --- | --- | --- |
| **N** | 4676-4792 | 3854-3928 |  |  |  |
| **Sex (males)** | 2386 (49.67%) | 1961 (49.8%) | 0.005 | 1 | 0.9 |
| **Zygosity (monozygotic twins)** | 1498 (31.18%) | 1256 (31.9%) | 0.469 | 1 | 0.5 |
| **Ethnicity (white)** | 4178 (86.99%) | 3478 (88.2%) | 3.15 | 1 | 0.08 |
| **Gestational Age (in weeks)** | 36.2 (2.48) | 36.2 (2.5) | 0.1125 | 8710 | 0.9 |
| **Weight at Birth (in kg)** | 2.46 (0.54) | 2.5 (0.5) | 0.3491 | 8508 | 0.7 |
| **Maternal Age at Birth** | 32.95 (5.19) | 33.3 (5.0) | 3.6039 | 8724 | <0.01 |
